# Modeling the effects of vector control interventions in reducing malaria transmission, morbidity and mortality

**DOI:** 10.1186/1475-2875-9-S2-O7

**Published:** 2010-10-20

**Authors:** Nakul Chitnis, Diggory Hardy, Guillaume Gnaegi, Konstantina Boutsika, Nicolas Maire, Richard Steketee, Allan Schapira, Tom Smith

**Affiliations:** 1 Department of Epidemiology and Public Health, Swiss Tropical and Public Health Institute, P.O. Box, CH-4002 Basel, Switzerland; 2 University of Basel, P.O. Box, CH-4003 Basel, Switzerland; 3 MACEPA-PATH, 01210 Ferney Voltaire, France

## 

Malaria interventions are usually prioritized using efficacy estimates from intervention trials, without considering the context of existing intervention packages or long-term dynamics. We use numerical simulation of mathematical models of malaria in humans and mosquitoes to provide robust quantitative predictions of effectiveness of different strategies in reducing transmission, morbidity and mortality.

We can simulate indoor residual spraying (IRS) and insecticide-treated nets (ITNs), used singly and in combination with each other and with other interventions such as improved case management, intermittent preventive treatment (IPT). We can estimate reductions in entomological inoculation rate (EIR), clinical cases, prevalence and malaria deaths from simulations of different coverage levels ITNs and IRS with different properties, and at different transmission and health system settings.

**Figure 1:**
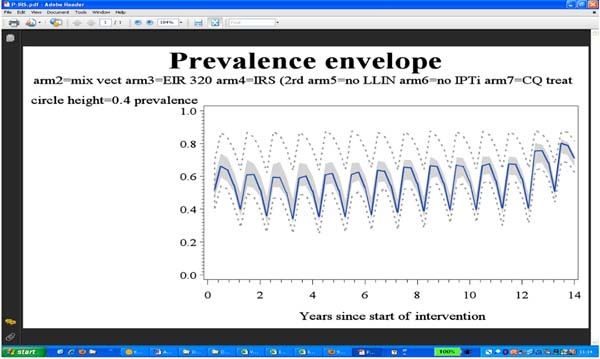
Model predictions of the effect of IRS with DDT on malaria prevalence. We assume two annual IRS DDT spray rounds (each with 95% coverage) for 12 years; simulations are of 1000 humans exposed to seasonal transmission based on a Tanzanian setting, with an initial EIR of 320 infectious bites per person per annum. The blue line is the median prevalence from four runs of each of 15 different model paramerizations for malaria in humans; grey area: interquartile range; dashed lines: maxima and minima.

Our results suggest that sustained coverage of one or two interventions reduces malaria prevalence in two to three years but does not lead to further gains (Figure 1). However, in some settings, even with sustained coverage, clinical incidence of malaria increases as the population loses its naturally acquired immunity. In some low to medium transmission settings, our simulations suggest that high coverage of both interventions can lead to interruption of transmission.
